# Quantification and Localization of Formylated Phloroglucinol Compounds (FPCs) in *Eucalyptus* Species

**DOI:** 10.3389/fpls.2019.00186

**Published:** 2019-02-26

**Authors:** Bruna Marques dos Santos, Juliane F. S. Zibrandtsen, Disan Gunbilig, Mette Sørensen, Federico Cozzi, Berin A. Boughton, Allison Maree Heskes, Elizabeth Heather Jakobsen Neilson

**Affiliations:** ^1^Section for Plant Biochemistry, Department of Plant and Environmental Sciences, University of Copenhagen, Copenhagen, Denmark; ^2^VILLUM Center for Plant Plasticity, Department of Plant and Environmental Sciences, University of Copenhagen, Copenhagen, Denmark; ^3^Section for Molecular Plant Biology, Department of Plant and Environmental Sciences, University of Copenhagen, Copenhagen, Denmark; ^4^School of BioSciences, University of Melbourne, Parkville, VIC, Australia; ^5^Metabolomics Australia, School of BioSciences, University of Melbourne, Parkville, VIC, Australia; ^6^Center for Synthetic Biology ‘bioSYNergy’, Department of Plant and Environmental Sciences, University of Copenhagen, Copenhagen, Denmark

**Keywords:** *Corymbia*, *Eucalyptus*, formylated phloroglucinol compounds, macrocarpal, MALDI-mass spectrometry imaging, sideroxylonal, specialized metabolites

## Abstract

The *Eucalyptus* genus is a hyper-diverse group of long-lived trees from the Myrtaceae family, consisting of more than 700 species. *Eucalyptus* are widely distributed across their native Australian landscape and are the most widely planted hardwood forest trees in the world. The ecological and economic success of *Eucalyptus* trees is due, in part, to their ability to produce a plethora of specialized metabolites, which moderate abiotic and biotic interactions. Formylated phloroglucinol compounds (FPCs) are an important class of specialized metabolites in the Myrtaceae family, particularly abundant in *Eucalyptus*. FPCs are mono- to tetra-formylated phloroglucinol based derivatives, often with an attached terpene moiety. These compounds provide chemical defense against herbivory and display various bioactivities of pharmaceutical relevance. Despite their ecological and economic importance, and continued improvements into analytical techniques, FPCs have proved challenging to study. Here we present a simple and reliable method for FPCs extraction, identification and quantification by UHPLC-DAD-ESI-Q-TOF-MS/MS. The method was applied to leaf, flower bud, and flower samples of nine different eucalypt species, using a small amount of plant material. Authentic analytical standards were used to provide high resolution mass spectra and fragmentation patterns. A robust method provides opportunities for future investigations into the identification and quantification of FPCs in complex biological samples with high confidence. Furthermore, we present for the first time the tissue-based localization of FPCs in stem, leaf, and flower bud of *Eucalyptus* species measured by mass spectrometry imaging, providing important information for biosynthetic pathway discovery studies and for understanding the role of those compounds *in planta*.

## Introduction

The *Eucalyptus* genus (Myrtaceae) is composed of long-lived trees that dominate a vast array of climatic regions across Australia. *Eucalyptus* trees possess several traits which have made some species economically important, such as fast growth, good wood quality as well as disease and insect resistance (Grattapaglia et al., 2012). They are now grown all over the world across a diverse range of climates, providing renewable resources to produce essential oil, paper, pulp, timber, and other biomaterials. The biogeographical diversity and success of those plants are partly due to their ability to produce a plethora of specialized metabolites, such as terpenes, flavonoids, cyanogenic glucosides, and phloroglucinols. These compounds play an important role in moderating interactions with the environment and combating biotic and abiotic stresses.

Phloroglucinols are an important class of specialized metabolites widely distributed in different natural sources such as plants, marine organisms and microorganisms. In recent years, phloroglucinol derivatives, especially formylated phloroglucinol compounds (FPCs) have been a hot research topic due to their structurally interesting skeletons and important bioactivities including antimicrobial (Faqueti et al., 2015), anticancer (Qin et al., 2016) and antimalarial effects (Bharate et al., 2006). Accordingly, large efforts have been made to characterize new FPCs structures from plants (e.g., Shang et al., 2016; Liu et al., 2018; Qin et al., 2018). FPCs are mono to tetra-formylated phloroglucinol based derivatives often with an attached terpene moiety that occur in the Myrtaceae family, primarily in *Eucalyptus* species (Eschler et al., 2000). Due to the terpenoid moiety, FPCs have also been called formyl phloroglucinol meroterpenoids (FPMs) (Shang et al., 2016), but herein we refer to them as FPCs.

**Figure 1:**
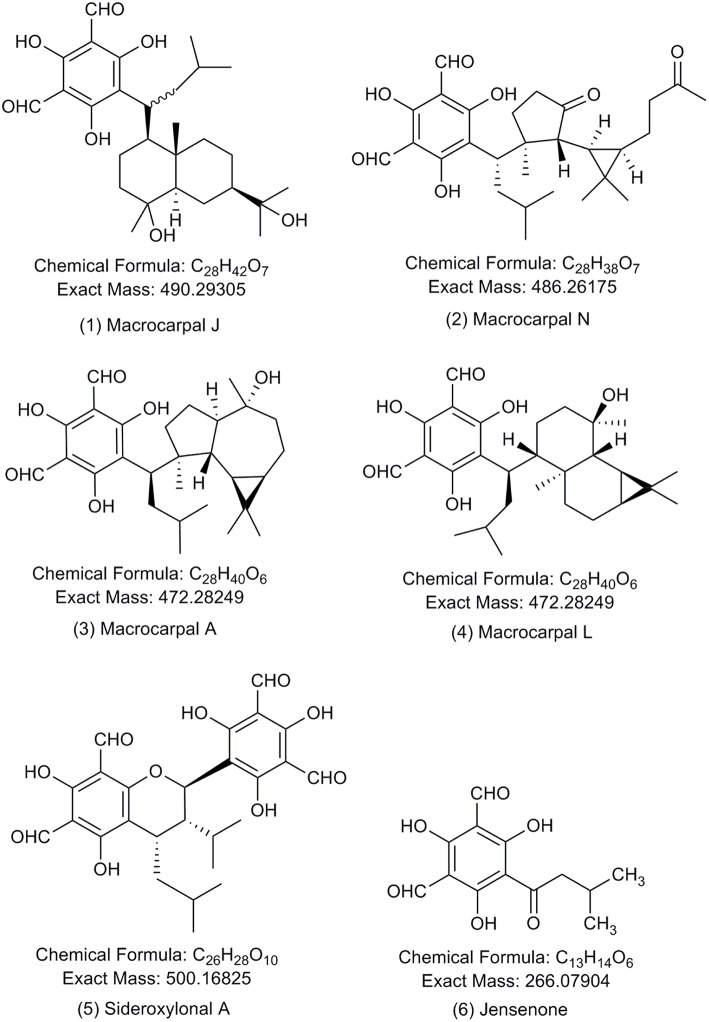
Chemical structures of the formylated phloroglucinol compounds (FPCs). Authentic analytical standards for compounds **1–5** were used in this study. Number of each compound corresponds to text and other figures.

The simplest FPCs are fully substituted, formylated acylphloroglucinols, such as jensenone (Figure 1). The units of jensenone form the basis of dimeric acylphloroglucinols, such as sideroxylonals and grandinal. The formylated acylphloroglucinols can also form adducts with mono- and sesqui-terpenes, such as euglobals and macrocarpals (Eschler et al., 2000; Moore et al., 2004b). The first FPC characterized was grandinol, isolated from *Eucalyptus grandis* and described as a root inhibitor (Crow et al., 1977). Since this first discovery, the *Eucalyptus* genus has proven to be a rich source of FPCs, with more than 70 compounds characterized in 39 species, predominantly in the subgenus *Symphyomyrtus* (Supplementary Table S1), with macrocarpals and sideroxylonals being the most common groups of FPCs reported in this genus (Moore et al., 2004b).

Sideroxylonals are compounds with a 2-phenylchromane skeleton. The typical structural characteristics of those compounds are the four formyl groups located in the aromatic rings at the positions C-3, C-5, C-3', and C-5', an isobutyl at C-7 and the isopropyl substituent is at C-10'. The differences between individual sideroxylonals appear in the stereochemistry at C-7 and C-10' (Sidana et al., 2010). There are three characterized sideroxylonals from *E. sideroxylon* and *E. melliodora* (Supplementary Table S1).

The macrocarpals possess an unusual skeleton that can be divided in two domains: one domain comprising a phloroglucinol dialdehyde moiety (common to all macrocarpals) and a second terpenoid domain (Alliot et al., 2013). Macrocarpal A (Murata et al., 1990) was the first macrocarpal to be isolated from *E. macrocarpa* and has its structure elucidated, showing an globulol skeleton in the terpenoid moiety. Seventeen other macrocarpals have since been isolated from various *Eucalyptus* species (Supplementary Table S1).

More recently, other species from the Myrtaceae family were discovered to be an abundant source of FPCs, with guava (*Psidium guajava*), *Rhodomyrtus* spp. and *Eugenia* spp. possessing 34, 7, and 4 new compounds, respectively (Supplementary Table S1). Despite their prevalence in the Myrtaceae family, especially in the important *Eucalyptus* genus, little is known about the biosynthesis and role of FPCs *in planta*.

Whilst several biosynthetic pathways for FPCs have been proposed (Yang et al., 2007; Yin et al., 2007; Shao et al., 2012; Gao et al., 2013; Jian et al., 2015; Qin et al., 2016; Tang et al., 2017), no formal studies have been carried out. Localization techniques, such as matrix assisted laser desorption/ionization mass spectrometry imaging (MALDI-MSI), can provide important information of specific cell or tissue localization of specialized metabolites, which can aid pathway discovery and give insight into the metabolite function (Boughton et al., 2016; Andersen et al., 2017; Heskes et al., 2018). To date, the only documented ecological role of FPCs is the strong deterrent effect against marsupial herbivores (Lawler et al., 2000; Moore et al., 2005) and insects (Matsuki et al., 2011). The limited work to investigate the role FPCs *in planta* is likely hindered by challenges related to identification and quantification. Furthermore, chemical synthesis studies have attempted to produce FPCs (Singh et al., 2010), however this is a difficult and costly process. Consequently, few analytical standards are available in the market, and these are obtained from the isolation and purification from many kilograms of *Eucalyptus* leaves.

The most widely cited method for extraction and quantitative determination of FPCs was described 15 years ago using HPLC-UV detection at 275 nm (Wallis et al., 2003). This is surprising considering the fast evolution of analytical chemistry techniques and significantly increased performance over this time. High-performance liquid chromatography, particularly when coupled with tandem mass spectrometry (HPLC-MS/MS), is the most suited method for the analysis of complex mixtures of phenolic components from vegetal origin due to high-sensitivity and specificity (Santos et al., 2013). The fragmentation pattern generated by high resolution tandem mass spectrometry is extremely valuable, because it can guide the identification or differentiation of structurally related compounds (Neilson et al., 2011; Heskes et al., 2012). Recently, HPLC-MS/MS allowed the putative identification of 13 FPCs among 70 phytoconstituents in an *E. sideroxylon* leaf extract (Okba et al., 2017), however the method was not developed specifically for FPCs, and may underestimate the diversity of this class of specialized metabolites. Here we present a fast, simple, and reliable method for FPCs extraction, detection, and quantification from complex biological samples using ultra-high-performance liquid chromatography coupled to ultra-violet diode array detection and electrospray ionization quadrupole time-of-flight tandem mass spectrometry (UHPLC-DAD-ESI-Q-TOF-MS/MS) system, which enabled the detection of 49 FPCs in one single leaf extract. Furthermore, to our knowledge we report for the first time the tissue-based localization of FPCs in leaf, stem and flower bud of two *Eucalyptus* species. Finally, a literature review of all characterized FPCs is presented.

## Materials and Methods

### Plant Material

Samples of the following eight *Eucalyptus* and one *Corymbia* species were harvested around Melbourne, VIC, Australia: *E. camphora* ssp*. humeana, E. camaldulensis, E. cladocalyx, E. leucoxylon, E. sideroxylon, E. viminalis, E. yarraensis*, and *C. ficifolia*. All *Eucalyptus* species belong to the subgenus *Symphyomyrtus*. All samples were harvested in biological triplicates from adult trees, kept on ice during transport to the laboratory and stored in −80°C until further analysis.

### Species Details and Tree Locations

*E. camaldulensis* (river red gum) is a large white-flowered tree widely distributed across Australia. Samples of leaves were harvested at the University of Melbourne (37°47'21.4”S 144°57'24.9”E) and in Royal Park (37°46'57.7”S 144°56'32.7”E) on the 4^th^ of December 2015 and the 9^th^ of March 2016, respectively.

*E. camphora* ssp*. humeana* (mountain swamp gum; henceforth referred to as *E. camphora*) is a small to medium-sized white flowered tree of south-east Australia. Samples of leaves, flower buds, and flowers were harvested in Buxton at Maroondah Highway by the Igloo Road House (37°25'22.8”S, 145°42'32.4”E) on the 6^th^ of January 2015.

*E. cladocalyx* (sugar gum) is a small to medium-sized white-flowered tree of South Australia. Samples of leaves, flower buds, and flowers were harvested at the University of Melbourne (37°79'66.0'S, 144°95'96.4'E), in the Royal Botanic Garden (37°83'26.7'S, 144°98'28.7'E) and at Creswick Campus 37°42'25.8'S, 143°89'92.7'E) on the 23^rd^ of January 2015, 12^th^ of February 2015 and the 5^th^ of February 2015, respectively.

*E. globulus* (blue gum) is a large white-flowered tree endemic to south-east Australia and Tasmania. Leaves of tree individual adult trees were harvested at the University of Melbourne, Creswick Campus (37°42'25.8'S, 143°89'92.7'E) on the 5^th^ of February 2015.

*E. leucoxylon* (yellow gum) is a small to medium-sized pink, red or yellow-flowered tree of south-east Australia, Kangaroo Island, Flinders Ranges, and Mount Lofty Range. Samples of leaves and flowers were harvested in Royal Park (a pink-flowered individual at 37°47'21.4”S 144°57'24.9”E) and at Monash University, Clayton Campus (a bright yellow-flowered individual at 37°54'24.5”S 145°08'.9”E and a red-flowered individual at 37°54'28.2”S 145°08'24.1”E) on the 27^th^ and 30^th^ of November 2015, respectively.

*E. sideroxylon* (red ironbark) is a medium to large-sized white, pink, red, or light yellow-flowered tree naturally distributed in east and south-east Australia. Samples of leaves and flowers were harvested in Princes Park (two creamy/white-flowered individuals at 37°47'21.4”S 144°57'24.9”E) and at Monash University, Clayton Campus (a pink-flowered individual at 37°54'26.7”S 145°08'18.0”E) on the 27^th^ and 30^th^ of November 2015, respectively.

*E. viminalis* (manna gum) is a large white-flowered tree common in New South Wales and Victoria, on Phillip Island and Tasmania. Samples of leaves and flowers were harvested on Phillip Island (38°29'04.4”S 145°15'56.2”E) on the 16^th^ of February 2016.

*E. yarraensis* (Yarra gum) is a medium-sized white-flowered tree of south-eastern Australia. Samples of adult leaves and flower buds were harvested in Coldstream at Maroondah Highway (37°73'46.2'S, 145°37'66.4'E) on the 6^th^ of January 2015.

*Corymbia ficifolia* (red-flowering gum) is a small to medium-sized red-flowered tree of south-west of Western Australia. Samples of leaves and flowers were harvested at the University of Melbourne (37°47'46.7”S 144°57'33.0”E) and at Monash University, Clayton Campus (37°54'50.3”S 145°07'48.3”E) on the 12^th^ and 13^th^ of January 2016, respectively.

### Solvents and Chemicals

Acetonitrile and methanol of HPLC-grade with purity ≥99.9% were purchased from Sigma Aldrich (Schnelldorf, Germany). Pierce^TM^ LC-MS grade formic acid was purchased from Thermo Fisher Scientific. Sodium formate for internal mass calibration of each analysis was purchased from Sigma Aldrich (Steinheim, Germany). FPCs analytical standards were purchased from BOC Sciences (NY, USA) for macrocarpal J (1), macrocarpal N (2), macrocarpal A (3), macrocarpal L (4), and sideroxylonal A (5) shown in Figure 1. Care must be taken when sourcing commercial FPCs standards. In our experience, so called authentic standards provided by a prominent supplier were determined in our laboratory by standard analytical chemistry techniques (^1^H, ^13^C NMR, and MS/MS) to be other unrelated chemical compounds and not suitable for the purpose.

### Extraction Procedure

Frozen plant material was ground in liquid nitrogen with mortar and pestle and ~100 mg was weighed in a 2 mL screw-lid Eppendorf tube. The plant material was boiled in 500 μL of extraction solvent (85% methanol and 0.1% formic acid in water) for 5 min and immediately cooled on ice for 10–15 min. Subsequently, the samples were centrifuged (15,000 × *g*, 5 min at 4°C) and the supernatant transferred into a new brown glass vial and stored at −80°C until further analysis. The plant material was oven-dried for 24 h at 70°C and the dry weight was recorded. Before the LC-MS analysis, samples were diluted 5 times in 0.1% formic acid in water and filtered through a membrane filter (0.45 μm, Merck Millipore).

### Preparation of Standard Curve

Authentic FPCs standards were freshly dissolved in methanol (1 mg /mL) in brown glass vials and diluted in water to different concentrations, depending on the range of the calibration curve. The solubilized FPCs standards had a stable signal intensity for maximum 3 days. Therefore, all standards were freshly prepared and analyzed immediately.

### UHPLC-DAD- ESI-Q-TOF-MS/MS

Chromatographic separation was performed on a Dionex Ultimate 3000RS UHPLC (Thermo Fisher Scientific) system with DAD detector, cooling auto-sampler at 10°C and column oven at 40°C. For data acquisition and processing, Compass DataAnalysis software (version 4.3, Bruker Daltonics) was used. Five microliter of the extracts were separated using a Phenomenex Kinetex® column (150 × 2.1 mm) packed with 1.7 μm C18 material with pore size of 100Å. Extracts were eluted under gradient conditions at constant flow rate of 0.3 mL/min as follows: 50% solvent A (0.05% formic acid in water) and 50% solvent B (0.05% formic acid in acetonitrile) linearly increasing to 100% solvent B in 20 min, hold for 13 min, and finally decreasing to initial conditions and re-equilibrating the column for 10 min.

The UHPLC system was coupled to compact™ (Bruker Daltonics) mass spectrometer with an electrospray ionization source. Eluted compounds were detected from *m/z* 50–1200 in negative ion mode under the following instrument settings: nebulizer gas, nitrogen at 2 bar; dry gas, nitrogen at 8 L/min and 250°C; capillary, 4,500 V; in-source CID energy, 0 V; hexapole RF, 100 Vpp; quadrupole ion energy, 4 eV; collision gas, nitrogen; MS/MS bbCID collision energy, 20 eV; collision RF 500 Vpp; transfer time, 100 μs; prepulse storage, 5 μs; spectra rate, 1 Hz; number of precursor ions, 3; active exclusion, 3 spectra, exclusion release, 12 s. Direct infusion of 10 mM sodium formate in the mass spectrometer at the beginning of the LC-MS run using a syringe pump setup with flow rate of 3 μL/min was performed to allow internal mass calibration of each analysis. All samples and standards were run in full scan mode for accurate quantification, and subsequently ran in autoMS/MS mode to acquire fragmentation information for the correct identification of FPCs.

Raw data was processed using the software DataAnalysis 4.2 from Bruker Daltonics. Automatic internal calibration was applied for each sample using the negative ions of the sodium formate cluster, in high precision calibration mode, search range *m/z* of 0.05 and intensity threshold of 1,000. Extracted ion chromatograms (EIC) for specific [M-H]^−^ ions were used to locate compounds. The identification of FPCs was based on the UV absorbance at 275 nm, measured [M-H]^−^ with < ± 2 ppm error when compared to the accurate [M-H]^−^ and the presence of the diagnostic fragment ions *m/z* 249, 207, and 181 observed in the authentic analytical standards. Calibration curves covered the range 0.5–75 μM (Supplementary Table S3) and were used for absolute quantification of the five compounds corresponding to the analytical standards. Other macrocarpals were relatively quantified using the calibration curve for macrocarpal A (3). Other sideroxylonals were relatively quantified using the calibration curve for sideroxylonal A (5). The sum of the peak areas at *m/z* 485.2557, 489.2858, 471.2752, and 499.1610 were used for the relative quantification of total FPCs.

### Statistical Analysis

Total FPCs concentration was statistically analyzed in SigmaPlot for Windows version 13.0 using One-Way ANOVA. The dataset was square root transformed to meet the normality and equal variance assumptions.

### Sample Preparation for MALDI-MSI

To locate FPCs in the different tissues, matrix-assisted laser desorption/ionization mass spectrometry imaging (MALDI-MSI) was carried out according to the procedure described in Schmidt et al. (2018). In short, the plant material was embedded in denatured albumin (boiled egg-white) and gently frozen over a bath of liquid nitrogen. Albumin embedded tissue was cryo-sectioned (30 μm) (Leica CM3050 S cryostat, Leica Microsystems, Wetzler, Germany), mounted onto double-sided carbon tape attached to glass slides and freeze-dried overnight. 2,5-Dihydroxybenzoic acid (DHB) was used as matrix and sublimed onto tissue sections using a custom-built sublimation apparatus. MS images were acquired in positive ion mode using a Bruker SolariX 7-Tesla Hybrid ESI/MALDI-FT-ICR-MS (Bruker Daltonics, Bremen, Germany).

A mass range of 100–2,000 *m/z* was employed with the instrument set to broadband mode with a time domain for acquisition of 2M providing an estimated resolving power of 130,000 at *m/z* 400. The laser was set to 30–45% power using the minimum spot size (laser spot size ~10 × 15 μm), smart-walk selected and random raster enabled resulting in ablation spots of ~35–40 μm in diameter, a total 1,500–2,000 shots were fired per spectra (pixel) at a frequency of 2 kHz within a 40 × 40 μm array. Optical images of tissue sections were acquired using an Epson Photosmart 4,480 flatbed scanner using a minimum setting of 4,800 d.p.i. Optical images are presented as an inverted image, generated in Adobe Photoshop CS2 (Adobe). The data were analyzed using Compass FlexImaging 4.1 (Bruker Daltonics), data was normalized using RMS normalization and individual ion images were scaled as a percentage of maximum signal intensity to enhance visualization. Potassium adducts of sideroxylonals [[M + K]^+^ *m/z* 539.1314, 0.01 ppm error] and other FPCs [[M + K]^+^ *m/z* 493.2351, −0.04 ppm error, *m/z* 511.2456, 0.09 ppm error, and *m/z* 525.2249, 0.02 ppm error]. A full list of FPCs corresponding to these specific masses is presented in the Supplementary Table S1.

## Results

### Detection and Quantification of FPCs

**Figure 2:**
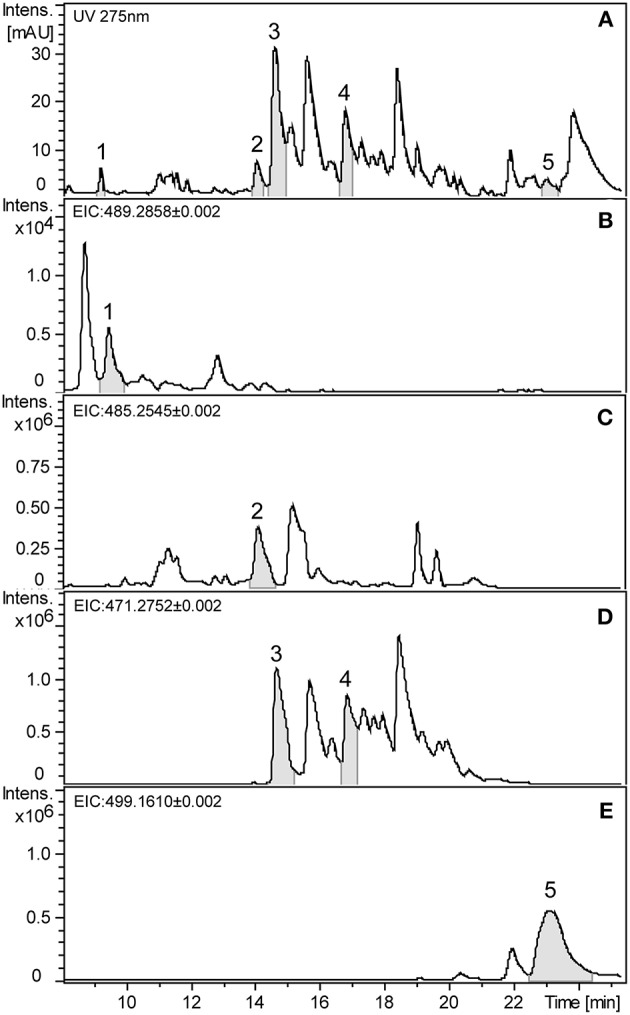
Representative chromatograms of *E. globulus* leaf extract showing FPCs. **(A)** UHPLC-UV chromatogram at 275 nm. **(B)** Extracted ion chromatogram (EIC) in negative mode of the *m/z* 489.2858 showing macrocarpal J (**1**). **(C)** EIC of the *m/z* 485.2545 showing macrocarpal N (**2**). **(D)** EIC of the *m/z* 471.2752 showing macrocarpal A (**3**) and L (**4**). **(E)** EIC of the *m/z* 499.1610 showing sideroxylonal A (**5**).

Formylated phloroglucinol compounds (FPCs) were successfully extracted, detected, and quantified from different *Eucalyptus* tissues by UHPLC-DAD-ESI-Q-TOF-MS/MS. Representative UV chromatogram and extracted ion chromatograms from *E. globulus* leaves are shown in Figure 2. The maximal absorbance UV wavelength of 275 nm is commonly used to detect FPCs (Eschler et al., 2000), therefore in Figure 2A it is possible to see a peak for each of the five analytical standards highlighted, but also many other peaks with the same *m/z* value. In general, FPCs require a high concentration of organic solvent to be eluted from C18 columns, and in this study the first FPC peak elutes after ~7.5 min, at 68% solvent B showing *m/z* value of 489.2861. The last compound eluted is sideroxylonal A, at ~23.3 min, with 100% solvent B.

**Figure 3:**
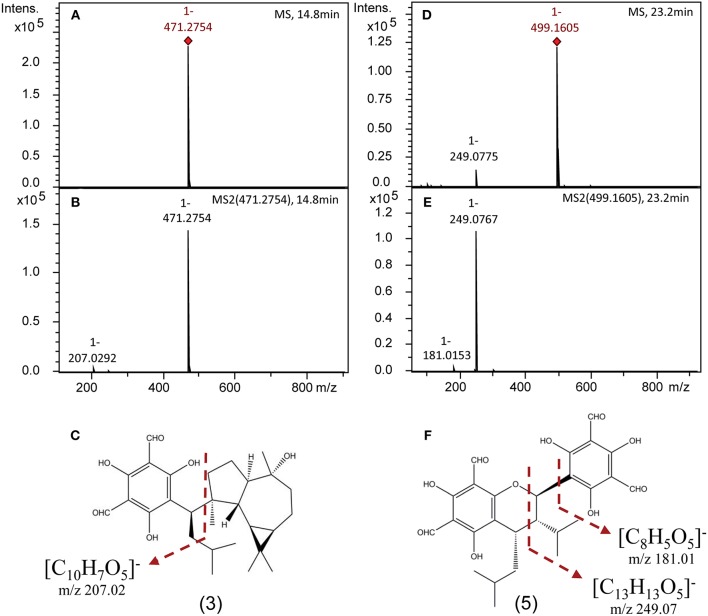
Representative negative ion mode mass spectra of FPCs based on authentic analytical standards obtained on a ESI-Q-TOF-MS/MS. **(A)** Full scan mass spectrum [M-H]^−^ of **3**. **(B)** Tandem MS (MS^2^) spectrum of **3**. **(C)** Structural position of the diagnostic fragment for macrocarpals, *m/z* 207. **(D)** Full scan mass spectrum [M-H]^−^ of **5**. **(E)** Tandem MS (MS^2^) spectrum of **5**. **(F)** Structural position of the diagnostic fragments for sideroxylonals, *m/z* 249 and 181.

Authentic analytical standards of compounds **1-5** were subjected to MS/MS and characteristic fragmentation patterns identified (Figure 3). For the macrocarpals, compounds **1–4**, the most common and intense fragment is the ion *m/z* 207, which was subsequently used as a diagnostic fragment ion for this group of FPCs. For compound **5**, the ions *m/z* 249 and 181 were the most intense and subsequently used as diagnostic fragments for sideroxylonals.

A total of 49 peaks were detected with characteristic FPCs features (Table 1). Identification was based upon the combination of UV absorbance at 275 nm, comparison of measured [M-H]^−^ with theoretical [M-H]^−^ within < ± 2 ppm error and the presence of the diagnostic fragments for FPCs. Among all peaks detected, 32 were phloroglucinol-sesquiterpene-coupled compounds, corresponding to the *m/z* value of 471 such as compounds **1** and **2**, *m/z* 489 such as compound **3**, and *m/z* 485 such as compound **4** (Figure 1). Four peaks corresponded to phloroglucinol dimers with an *m/z* value of 499, such as compound **5**, jensenal, grandinal, and sideroxylonal B and C. The peak with *m/z* 451.2497 was putatively identified as eucalyptal D (C_28_H_36_O_5_), as there are no other FPCs described that would correspond to this *m/z* value. Finally, nine peaks with *m/z* 453 and three peaks with *m/z* 467, also corresponded to phloroglucinol-sesquiterpene-coupled compounds (Supplementary Table S1).

**Table 1:** FPCs detected in methanol extracts of *Eucalyptus* samples (leaf, flower bud, and flower) analyzed by UHPLC-DAD-ESI-Q-TOF-MS/MS.

**Compound**	**Molecular formula**	**Accurate [M-H]^**−**^**	**Observed [M-H]^**−**^**	**Error (ppm)**	**RT (min)**	**Abundant MS/MS fragments**	***Eca***	***Ec***	***Eg***	***El***	***Es***	***Ev***	***Ey***
FPC 1	C28H42O7	489.2858	489.2861	−0.66	7.4	207, 249, 237, 282	n.d.	L, FB	L	L, F	L, F	L	FB
FPC 2	C28H38O7	485.2557	485.2557	0	8.4	207, 250, 291	n.d.	L	L	F	n.d.	F	n.d.
FPC 3	C28H42O7	489.2858	489.2866	−1.68	8.8	207, 249, 237, 282	L	L, FB	L	L, F	L, F	L	FB
Macrocarpal J	C28H42O7	489.2858	489.2852	1.18	9.5	489, 207	L	L, FB	L	L, F	L, F	n.d.	n.d.
FPC 4	C28H38O7	485.2557	485.2553	0.82	9.6	207, 250, 291	L	L	L	n.d.	L, F	n.d.	n.d.
FPC 5	C28H38O7	485.2557	485.255	1.44	10.1	207, 250, 291	L	L, F	L	F	L, F	L	n.d.
FPC 6	C28H38O7	485.2557	485.2555	0.41	10.7	207, 250, 291	L	L, F	L	n.d.	L	L	n.d.
FPC 7	C28H38O7	485.2557	485.2548	1.85	11.2	207, 250, 291	L	L, FB, F	L	L, F	L	F	n.d.
FPC 8	C28H38O7	485.2557	485.2548	1.85	11.5	207, 250, 291	L	L, FB	L	F	L, F	L	n.d.
FPC 9	C28H38O7	485.2557	485.2555	0.41	11.7	207, 250, 291	L	L, F	L	L	L, F	F	n.d.
FPC 10	C28H38O7	485.2557	485.2555	0.41	12.8	207, 250, 291	L	L, F	L	L, F	L	L	n.d.
FPC 11	C28H38O7	485.2557	485.2551	1.24	13.2	207, 250, 291	L	L, F	L	L, F	F	F	n.d.
Macrocarpal N	C28H38O7	485.2545	485.2542	0.57	13.9	485, 457, 250, 207	L	L, FB, F	L	L, F	L, F	L, F	n.d.
FPC 12	C28H40O6	471.2752	471.2756	−0.82	14.7	207, 250, 282, 383	L	L, FB, F	L	L, F	L, F	L, F	FB
Macrocarpal A	C28H40O6	471.2752	471.2749	0.66	14.8	471, 250, 207	L	L, FB, F	L	L, F	L, F	L, F	L, FB
FPC 13	C28H38O7	485.2557	485.2551	1.24	15.4	207, 250, 291	n.d.	L, F	L	F	n.d.	F	n.d.
FPC 14	C28H40O6	471.2752	471.275	0.45	15.6	207, 250, 282, 383	L	L, FB, F	L	L, F	L, F	L, F	FB
FPC 15	C28H38O7	485.2557	485.255	1.44	15.7	207, 250, 291	L	L, FB	L	L, F	L, F	L	n.d.
FPC 16	C28H40O6	471.2752	471.2753	−0.18	15.8	207, 250, 282, 383	L	L, FB, F	L	L, F	L, F	L, F	FB
FPC 17	C28H38O7	485.2557	485.2556	0.21	16.1	207, 250, 291	L	L, FB, F	L	n.d.	n.d.	F	n.d.
FPC 18	C28H40O6	471.2752	471.275	0.45	16.5	207, 250, 282, 383	L	L, FB, F	L	L, F	L, F	L, F	FB
FPC 19	C28H40O6	471.2752	471.2754	−0.4	17	207, 250, 282, 383	L	L, FB, F	L	L, F	L, F	L, F	FB
Macrocarpal L	C28H40O6	471.2752	471.2748	0.88	17.1	471, 250, 207	L	L, FB, F	L	L, F	L, F	L, F	L, FB
FPC 20	C28H40O6	471.2752	471.2754	−0.4	17.4	207, 250, 282, 383	L	L, FB, F	L	L, F	L, F	L, F	n.d.
FPC 21	C28H40O6	471.2752	471.2755	−0.61	17.7	207, 250, 282, 383	L	L, FB, F	L	L, F	L, F	L, F	FB
FPC 22	C28H40O6	471.2752	471.2753	−0.18	18	207, 250, 282, 383	L	L, FB, F	L	L, F	L, F	L, F	n.d.
FPC 23	C28H36O6	467.2439	467.2446	−1.47	18.2	207, 250	L	L	L	L, F	L, F	L, F	n.d.
FPC 24	C28H40O6	471.2752	471.2754	−0.4	18.3	207, 250, 282, 383	L	L, FB, F	L	L, F	L, F	L, F	FB
FPC 25	C28H40O6	471.2752	471.2754	−0.4	18.5	207, 250, 282, 383	L	L, FB, F	L	L, F	L, F	L, F	n.d.
FPC 26	C28H38O7	485.2557	485.2556	0.21	19.1	207, 250, 291	L	L, FB, F	L	L, F	F	F	L, FB
FPC 27	C28H40O6	471.2752	471.2753	−0.18	19.2	207, 250, 282, 383	L	L, FB, F	L	L, F	L, F	L, F	FB
FPC 28	C28H40O6	471.2752	471.2754	−0.4	19.6	207, 250, 282, 383	L	L, FB, F	L	L, F	L, F	L, F	FB
FPC 29	C28H38O7	485.2557	485.2549	1.65	19.7	207, 250, 291	L	L, FB, F	L	n.d.	F	F	FB
FPC 30	C28H40O6	471.2752	471.2752	0.03	20	207, 250, 282, 383	L	L, FB, F	L	L, F	L, F	L, F	n.d.
FPC 31	C26H28O10	499.161	499.1619	−1.86	20.4	181, 249, 453	L	L, FB, F	L	L, F	L, F	L, F	n.d.
FPC 32	C28H38O5	453.2646	453.2649	−0.56	20.9	207, 250	L	L, F	L	F	L	F	n.d.
FPC 33	C28H36O5	451.249	451.2497	−1.56	21	207, 250	L	L, FB, F	L	L, F	L, F	L, F	n.d.
FPC 34	C28H38O5	453.2646	453.2648	−0.34	21.1	207, 250	L	L, F	L	F	L, F	F	n.d.
FPC 35	C28H36O6	467.2439	467.2448	−1.9	21.2	207, 250	L	L	L	L, F	L, F	L, F	n.d.
FPC 36	C28H38O5	453.2646	453.2648	−0.34	21.4	207, 250	L	L, FB, F	L	F	L, F	L, F	L
FPC 37	C28H38O5	453.2646	453.2648	−0.34	21.5	207, 250	n.d.	L, F	L	F	n.d.	F	n.d.
FPC 38	C28H38O5	453.2646	453.2648	−0.34	21.6	207, 250	n.d.	L, FB, F	L	F	F	F	n.d.
FPC 39	C28H38O5	453.2646	453.265	−0.78	21.7	207, 250	n.d.	L, F	L	F	n.d.	F	n.d.
FPC 40	C26H28O10	499.161	499.1615	−1.06	21.9	181, 249, 453	L	L, FB, F	L	L, F	L, F	L, F	n.d.
FPC 41	C28H38O5	453.2646	453.2648	−0.34	22	207, 250	L	L, FB, F	L	F	F	F	n.d.
FPC 42	C28H38O5	453.2646	453.2647	−0.11	22.3	207, 250	n.d.	L, F	L	L, F	F	L, F	L
FPC 43	C28H38O5	453.2646	453.2648	−0.34	22.5	207, 250	n.d.	L, FB, F	L	L, F	n.d.	L, F	L
FPC 44	C26H28O10	499.161	499.1613	−0.66	22.8	181, 249, 453	L	L, FB, F	L	L, F	L, F	L, F	n.d.
Sideroxylonal A	C26H28O10	499.161	499.1605	0.94	23.3	249, 181	L	L, FB, F	L	L, F	L, F	L, F	n.d.

*Eca, E. camaldulensis; Ec, E. camphora; Eg, E. globulus; El, E. leucoxylon; Es, E. sideroxylon; Ev, E. viminalis; Ey, E. yarraensis; n.d., not detected; L, leaf; FB, flower bud; F, flower. N.B. Only leaves were analyzed for E. camaldulensis and E. globulus*.

**Figure 4:**
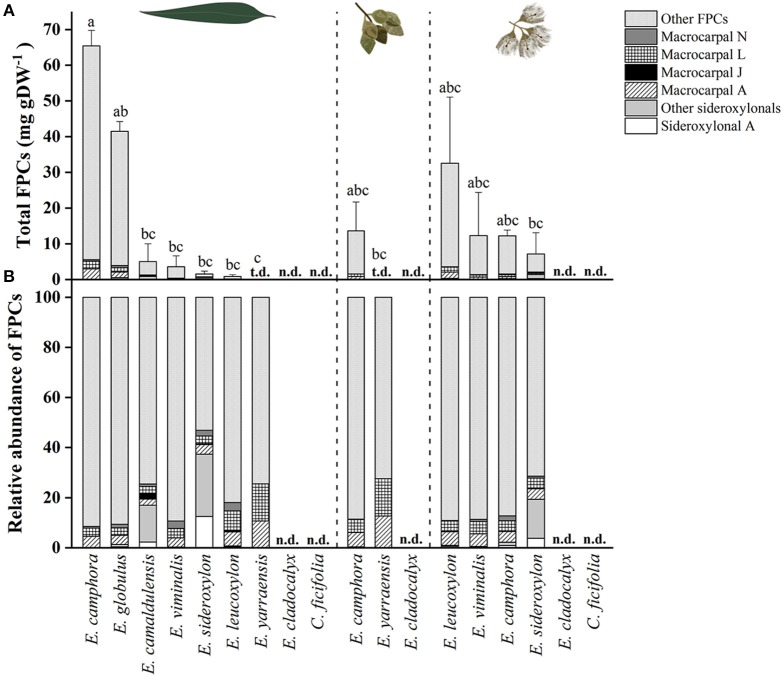
**(A)** Total FPCs concentration in leaves, flower buds and flowers of different eucalypt species. Bars represent mean ± standard error. Small letters represent statistical differences according to one-way ANOVA *p* < 0.050. **(B)** Relative FPCs concentration as percentage in respective tissues. t.d., traces detected; n.d., not detected.

From all species analyzed, *E. camphora* and *E. globulus* had the highest concentration of total FPCs in leaves, with 65 and 41 mg g^−1^ DW, respectively (Figure 4, Supplementary Table S2). *Eucalyptus camphora* also had high concentration of FPCs in flower buds and flowers, with 13 and 12 mg g^−1^ DW, respectively. Interestingly, three *Eucalyptus* species showed a tendency to accumulate more FPCs in flowers compared to the leaves. *Eucalyptus leucoxylon, E. sideroxylon*, and *E. viminalis* contained ~40, 5, and 3 times more total FPCs in the flowers compared to leaves, respectively Figure 4, Supplementary Table S2. *Eucalyptus yarraensis* presented very low amounts of FPCs in leaves and flower buds, and it is the only species that does not contain any sideroxylonals. *Eucalyptus cladocalyx* and *C. ficifolia* did not show any traces of this class of specialized metabolites in the tissues analyzed.

### Localization of FPCs in *Eucalyptus* Tissues by MALDI-MSI

**Figure 5:**
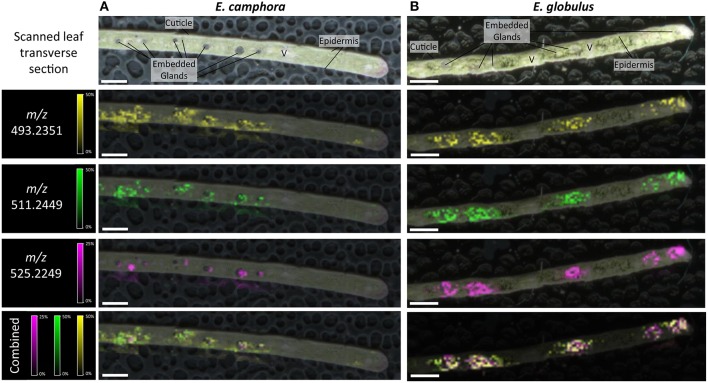
Localization of FPCs in *Eucalyptus camphora* **(A)** and *E. globulus* leaves **(B)**. Leaf transverse sections were prepared for matrix-assisted laser desorption ionization-mass spectrometry imaging (MALDI-MSI). Corresponding ion maps of different FPCs are shown: yellow; *m/z* 493.2351; [M+K]^+^, green *m/z* 511.2449; [M+K]^+^, pink *m/z* 525.2249; [M+K]^+^, and a combination of all selected ions overlaid. FPCs are associated to the embedded glands in both species. Some FPC ions also localize to the epidermis. All images are root mean square (RMS) normalized, with internal scaling of 50% for the ions *m/z* 493.2351 and 511.2449, and 25% for *m/z* 525.2249. Scale bar = 500 μM. V, vascular tissue.

**Figure 6:**
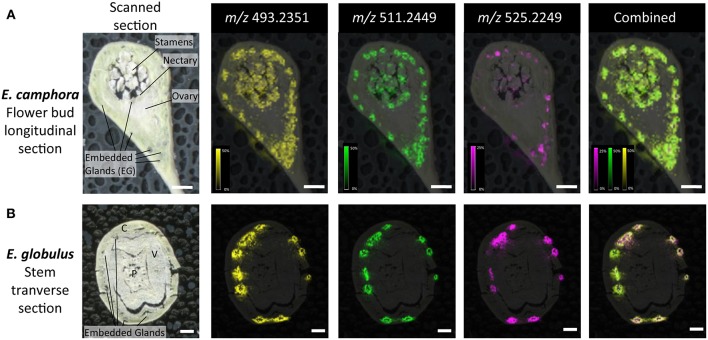
Localization of FPCs in *Eucalyptus camphora* flower bud **(A)** and *E. globulus* stem **(B)**. A longitudinal section of a flower bud and transverse section of a stem were prepared for matrix-assisted laser desorption ionization-mass spectrometry imaging (MALDI-MSI). Corresponding ion maps of different FPCs are shown: yellow; *m/z* 493.2351; [M+K]^+^, green *m/z* 511.2449; [M+K]^+^, pink *m/z* 525.2249; [M+K]^+^, and a combination of all selected ions overlaid. FPCs are associated to the embedded glands, adjacent to the epidermis of the flower bud, to the embedded glands beneath the nectary, and to the stamens. For the stems, FPCs are localized to embedded glands within the cortex. All images are root mean square (RMS) normalized, with internal scaling of 50% for the ions *m/z* 493.2351 and 511.2449, and 25% for *m/z* 525.2249. Scale bar = 500 μM. C, cortex; P, pith; V, vascular tissue.

The spatial distribution of FPCs in flower bud, leaf and seedling stem from two *Eucalyptus* species were investigated by matrix-assisted laser desorption ionization-mass spectrometry imaging (MALDI-MSI; Figures 5, 6). MALDI-MSI revealed that FPCs were associated with the subdermal embedded glands in all tissues analyzed. In addition, FPCs were located in the epidermal layer adjacent to some embedded glands in the leaf and present within the stamens of the *E. camphora* flower bud. Ions corresponding to sideroxylonals were not detected.

### Detailed Review of FPC Prevalence and Characterization

Due to their apparent prevalence and importance in *Eucalyptus* and other Myrtaceae species, we conducted a detailed review of all characterized FPCs (Supplementary Table S1). Since the characterization of grandinol from *E. grandis* in 1977 (Crow et al., 1977), an impressive number of 162 FPCs have been described with more than 80 compounds characterized in 39 *Eucalyptus* species, predominantly in the subgenus *Symphyomyrtus*. More recently, other plants from the Myrtaceae family where discovered to be abundant sources of FPCs, including 34 new FPCs identified from guava (*Psidium guajava*) (for example psiguajadials A-K by (Tang et al., 2017); for more references see Supplementary Table S1), seven identified from *Rhodomyrtus* spp. and four identified from *Eugenia* spp. The structural differences between the most common FPCs found in *Eucalyptus* species vs. the new compounds identified in other genus inside the Myrtaceae family are illustrated in the Supplementary Figure S4.

## Discussion

### An Improved Method to Detect and Quantify FPCs

The most cited method for extraction and quantitative determination of FPCs was described 15 years ago (Wallis et al., 2003) using only HPLC-UV detection at 275 nm, with some modifications for sideroxylonals (Wallis and Foley, 2005). Near infrared reflectance spectroscopy (NIRS) has also been used to quickly predict sideroxylonal concentration in leaves but it is non-specific. In this method, the light reflected when a sample is exposed to light in the near-infrared spectrum depicts the chemical bonds in the sample. These spectra have to be calibrated against reference values obtained by analyzing a portion of the samples using traditional analytical chemistry methods such as HPLC (Foley et al., 1998; Wallis et al., 2002). It is evident that analytical limitations have restricted previous investigations of FPCs to the quantification of sideroxylonals only. Therefore, the qualitative and quantitative variation in this very diverse group of specialized metabolites remains largely unknown and the total concentration of those compounds might have been underestimated.

Here we present a fast, simple, and reliable method for FPCs extraction, detection, and quantification from complex biological samples using an UHPLC-DAD-ESI-Q-TOF-MS/MS system. Unlike the previous methods, the sample extraction presented here is much simpler and faster and is suited for small amounts of plant material, such as a single leaf. This allows future studies to investigate the qualitative and quantitative variation of FPCs e.g., in leaves of an individual tree and during seedling development.

In this study, we show that FPCs require high amounts of organic solvent to be eluted from the C18 column, in agreement with previous reports (Eyles et al., 2003; Moore et al., 2004b). Sideroxylonal A, for example, can only be eluted after 3 min at 100% solvent B. This suggests that most of the typical methods used in untargeted metabolomics studies using reverse-phase columns would miss this compound. The chromatograms also show many peaks corresponding to the calculated accurate mass of FPCs, and the identification of those peaks as FPCs was possible due to the typical UV absorbance at 275 nm and the comparison with authentic analytical standards accurate mass and fragmentation pattern. This shows the high diversity of FPCs in complex *Eucalyptus* extracts.

A specific fragmentation pattern for macrocarpals and sideroxylonals, the most abundant groups of FPCs reported in *Eucalyptus* samples (Moore et al., 2004b), has been found in this study. The characteristic fragment ion at *m/z* 249 from compound **5** results from the retro-Diels-Alder cleavage of the molecular ion (Soliman et al., 2014). It is the most abundant and thus the diagnostic fragment ion for sideroxylonals, followed by a less abundant peak of *m/z* 181, suggested to be the diformyl phloroglucinol moiety by Chenavas et al. (2015). For macrocarpals, the diagnostic fragment ion detected in the compounds **1-4** is *m/z* 207, in agreement with previous reports (Eyles et al., 2003; Okba et al., 2017), and we here suggest it to be the isopentyl diformyl phloroglucinol moiety + C_2_H_2_. The fragmentation pattern and the high-resolution mass spectra shown here have great value for future studies allowing the confident identification of FPCs, which have probably been overlooked in many other plant species due to the challenges of detecting them. Our method provides a detection limit as low as 0.5 μM of FPCs in a very complex plant extract (standard curves available in excel file—Supplementary Material).

### Qualitative and Quantitative FPCs Variation in *Eucalyptus*

Here we present the comprehensive list of FPCs identified in *Eucalyptus* tissue. Our results show that FPCs are more abundant and diverse than previously reported. For example, 49 FPCs peaks were identified in a single *E. globulus* leaf extract, undoubtedly representing some new uncharacterized compounds. Previously, Okba et al. (2017) putatively identified 13 FPCs in a bulk leaf extract of *E. sideroxylon* using HPLC-MS/MS. Using the current method, we report 34 FPCs identified in leaves of the same species. This significant increase in the number of FPCs detected reflect how method optimization increases detection sensitivity. The phloroglucinol-sesquiterpene compounds with *m/z* 471 and 485 are the most frequently detected peaks, and potentially corresponding to the *m/z* value of many different isomeric FPCs, such as macrocarpals.

The presence and abundance of FPCs is a highly variable trait. There is considerable intraspecific variation in their concentration as reported by Lawler et al. (2000). In the present study, *E. globulus* had one of the highest concentrations of total FPCs, dominated by macrocarpals (Figure 4), while in the study by Eschler et al. (2000) macrocarpals and sideroxylonals were not detected in this species. Even among the three biological replicates of *E. sideroxylon* used in the present study, we identified an individual that had no FPCs, while the two others possessed significant concentrations of sideroxylonals and macrocarpals. A striking example of variation in FPC abundance is demonstrated by the mosaic trees *E. melliodora* and *E. sideroxylon*. These individual trees had higher FPC concentration in one single branch, conferring resistance to insect herbivory (Padovan et al., 2012).

*Eucalyptus camphora* and *E. globulus* presented high concentrations of total FPCs in expanded leaves, with 65 and 41 mg g^−1^ DW, respectively. These concentrations are in a similar range to previous reports. For example, the concentration of sideroxylonals have been reported to reach up to 52 mg g^−1^ DW in *E. melliodora* (Wallis et al., 2002) and up to 100 mg g^−1^ DW in *E. loxophleba* ssp. *lissophloia* (Wallis and Foley, 2005).

Prior to this study, FPCs had only been detected in the reproductive tissue on one other occasion, with sideroxylonal C identified from flowers of *E. albens* (Neve et al., 1999). Here we show the presence of FPCs in flowers of four additional *Eucalyptus* species, and for three of them (*E. leucoxylon, E. viminalis*, and *E. sideroxylon*), the concentration of FPCs shows a tendency to be higher in the flowers compared to mature leaves. Interestingly, tissue-specific qualitative variation was observed, with some FPCs only detected in the leaves, or only in the reproductive tissue (Table 1). These observations could suggest tissue-specific roles for different FPCs.

The absence of FPCs in *E. cladocalyx* and *C. ficifolia* may suggest that these species do not synthesize these compounds. It may also be possible, however, that the FPCs are synthesized in other tissues, or at a different ontogenetic stage. For example, FPCs have been detected in the wood of *E. globulus* and *E. nitens*, following a wounding event (Eyles et al., 2003) and in fine roots of 12 species from the subgenera *Symphyomyrtus* and *Eucalyptus* (Senior et al., 2016). Qualitative polymorphism has been recorded for FPCs (Padovan et al., 2012) and other specialized metabolites classes in *Eucalyptus* e.g. cyanogenic glucosides (Neilson et al., 2011). Interestingly, *E. cladocalyx* synthesizes high levels of the cyanogenic glucoside prunasin (Hansen et al., 2018), which could suggest an alternative chemical defense strategy for this species. *Corymbia* spp. have been reported to contain FPCs previously (Eschler et al., 2000). The absence of FPCs from *C. ficifolia* leaves may be due to the lack of embedded glands (Brooker and Nicolle, 2013). Given that we have shown that FPCs are localized to the embedded glands, it could suggest that *C. ficifolia* does not possess the structural cells to biosynthesize or store these metabolites.

### Localization and Possible Roles of FPCs in *Eucalyptus*

To provide further insights into FPC metabolism, we conducted MALDI-MSI on the two highest producing species, *E. camphora* and *E. globulus*. MALDI-MSI can greatly aid in the understanding of physiological roles and provide valuable information for biosynthetic pathway elucidation (Boughton et al., 2016; Heskes et al., 2018). Here we show that FPCs are associated with the subdermal embedded glands in all the tissues analyzed. Localization of other *Eucalyptus* specialized metabolites such as mono- and sesqui-terpenes, flavones and monoterpene glucose esters have been reported to be present in embedded glands (Goodger et al., 2010, 2016). Localization of specialized metabolites to storage structures is widely prevalent across the plant kingdom, largely as a way to prevent autotoxicity (Knudsen et al., 2018). Upon tissue disruption, such as that caused by herbivory, the compounds are released to provide a layer of chemical defense. Accordingly, the localization of FPCs to the embedded gland supports their role as chemical defense compounds. In the case of *Eucalyptus*, the content of embedded glands was shown to be in two phases leading to the suggestion that monoterpene glucose esters are spatially separated from the mono- and sesqui-terpenes within the lumen (Heskes et al., 2012). In the results presented here, spatial organization of different FPCs within embedded glands could also be suggested, as the ion *m/z* 525.2249 typically locates to the center of the gland lumen, whilst the ions *m/z* 493.2351 and *m/z* 511.2449 locate to the outer perimeter of the glands. Higher resolution imaging of these embedded glands could provide further insight into more complex spatial separation and localization of FPCs. Interestingly, FPCs also co-localized to the leaf epidermal layer of several glands. It could be speculated that this may provide a metabolite highway by which the lipophilic mono- and sesqui-terpenes could be emitted from the embedded gland to the atmosphere.

The presence of FPCs in the stamens of flower buds could suggest a possible role in defense against florivores, or other roles such as attracting specific pollinators. Given the presence of oil glands and FPCs directly below the nectary, it could be speculated that the FPCs may also be present in *Eucalyptus* nectar to either attract pollinators or deter nectar robbers. To our knowledge, no study has investigated the concentration of different specialized metabolites in *Eucalyptus* nectar, and this is an intriguing avenue of research to pursue.

Overall, the role FPCs play in *Eucalyptus* leaves has been strongly linked to chemical defense against marsupial folivores. For example, the concentration of total FPCs was the most important variable determining feeding by marsupial folivores on *Eucalyptus* species (Lawler et al., 2000; Moore et al., 2005; Jensen et al., 2015), and were implicated in habitat patchiness in Australian forests (Lawler et al., 2000). Interestingly, the highest concentrations of total FPCs in this study were found in four koala-preferred species: *E. camphora, E. globulus, E. camaldulensis*, and *E. viminalis* (Moore et al., 2004a; Higgins et al., 2011). This could reflect the ongoing “arms race” between plant and herbivore, where koalas can tolerate significant levels of these compounds. Indeed, koalas can tolerate up to 50 mg of sideroxylonal per gram of dry matter (equivalent to 5%) (Moore et al., 2004a) and show specific behavioral traits when dealing with eucalypt diets of varying toxicity (Marsh et al., 2007). The metabolism and detoxification of FPCs by the koala is currently unknown. FPCs can also determine patterns of damage by Christmas beetles in *Eucalyptus* plantations (Steinbauer and Matsuki, 2004; Andrew et al., 2007; Matsuki et al., 2011). Andrew et al. (2007) suggests that the potential for evolution by natural selection of sideroxylonal concentrations is not strongly constrained by growth costs and that both growth and defense traits can be successfully incorporated into breeding programs for plantation trees. Therefore, FPCs are important not only in natural forest ecology, but also for commercial *Eucalyptus* plantations.

### FPC Prevalence, Characterization, and Future Research Directions

In recent years, FPCs have become a hot research topic due to their structurally interesting skeletons and important bioactivities including antimicrobial (Sidana et al., 2010; Faqueti et al., 2015), anticancer (Qin et al., 2016), and antimalarial effects (Bharate et al., 2006). The wide reported bioactivities of FPCs were reviewed in depth by Brezáni and Šmejkal (2013).

FPCs were once believed to be exclusive to *Eucalyptus* species, but according to our literature review, 91 new compounds were discovered during the past 10 years, with 42 FPCs isolated from other species in the Myrtaceae family (Supplementary Table S1). Their structural differences and similarities are illustrated in the Supplementary Figure S4. The FPCs found in *Eugenia umbelliflora* contain, in the phloroglucinol part, both an aldehyde and a butyroyl chain (e.g., Eugenial C), whereas the analogous compound Macrocarpal G widely occurring in the *Eucalyptus* species contain two aldehyde substituents (Faqueti et al., 2015). Most FPCs found in *Rhodomyrtus* plants have the skeleton of the phloroglucinol coupled to an eudesmane (sesquiterpene) moiety (Shou et al., 2014) (e.g., Rhodomyrtal A), showing similarity to grandinol, the very first FPC characterized which was isolated from *Eucalyptus grandis*. *Psidium* meroterpenoids are a subgroup of FPCs, which are exclusively reported from the species *Psidium guajava*. Structurally, they are characterized by the presences of 3,5-diformyl-benzyl phloroglucinol moiety and a dihydropyran ring junction (Tang et al., 2017), e.g., Psiguajadial C, F, and K in the Supplementary Figure S4.

Plant species from the Myrtaceae family have been used as medicinal plants by native populations in Brazil, South Africa, Australia, and China for centuries. This could be partially explained by the recent discovery of FPCs in *Psidium* and *Eugenia* species and the broad range of bioactivity demonstrated by those compounds (Shao et al., 2010; Faqueti et al., 2012, 2015). Considering the challenges to detect and quantify those compounds, we hypothesize that FPCs are more widespread in the plant kingdom and have been overlooked so far. The improved method presented here will be invaluable for the continued exploration of FPCs prevalence.

It is evident that FPCs have important ecological functions based on their role in deterring herbivores. However, many aspects of FPC prevalence and their potential multifunctional roles across different ecosystems remains elusive. A simple, reliable method for FPC detection and quantification will be of great benefit for impending studies. Future work into FPC biosynthesis will also aid the understanding of the role and regulation of these compounds across the *Eucalyptus* genus and beyond. Furthermore, with atmospheric CO_2_ and temperatures increasing at unprecedented rates, it is important to investigate how different climatic factors will influence FPC concentrations, and the impact this may have on ecological and commercial systems. Previous studies have shown that environmental stresses related to climate change—such as elevated atmospheric CO_2_ and ozone—can increase specialized metabolite concentration in *Eucalyptus* (Gleadow et al., 1998; Kanagendran et al., 2018). Our improved method to detect and quantify FPCs will allow future studies to investigate how those compounds respond to environmental changes. An increase in the concentration of FPCs in *Eucalyptus* leaves will have significant implications in terms of the palatability of foliage and defense against herbivores, directly affecting the food chain.

## Concluding Remarks

Here we demonstrate a fast, simple, and reliable extraction method suitable for FPCs in combination with UHPLC, DAD and MS/MS analysis. For the first time, authentic analytical standards were used to provide high resolution mass spectra and fragmentation patterns, which have great value for the correct identification of those compounds in complex biological samples. This class of specialized metabolites has been overlooked likely due to the challenges related to their identification and quantification. Therefore, we believe that the results presented here will allow future studies to identify FPCs with high accuracy, which is essential for better understanding the role of those compounds *in planta*, particularly through ontogenetic development and in response to biotic and abiotic stresses. The impressively large amount of FPCs detected in flowers opens the debate for different roles of these compounds. FPCs are also considered very bioactive molecules with potential applications in the pharmaceutical industry. The tissue-based localization presented here provides important information on the spatial distribution of FPCs in *Eucalyptus*, and, can directly contribute to pathway discovery studies by providing target tissues for gene expression analyses such as transcriptomics.

## Data Availability

The raw data supporting the conclusions of this manuscript will be made available by the authors, without undue reservation, to any qualified researcher.

## Author Contributions

EHJN conceived the study and research plans. BMS, DG, MS, JFSZ, and FC performed the experiments. EHJN, AMH, and BAB supervised experimental work. BAB provided technical assistance. BMS and EHJK analyzed the data. BMS wrote the manuscript with contributions from MS and EHJN. All authors have reviewed and approved the final manuscript.

### Conflict of Interest Statement

The authors declare that the research was conducted in the absence of any commercial or financial relationships that could be construed as a potential conflict of interest.
