# Corrigendum: Quantification and Localization of Formylated Phloroglucinol Compounds (FPCs) in *Eucalyptus* Species

**DOI:** 10.3389/fpls.2019.01052

**Published:** 2019-08-28

**Authors:** Bruna Marques dos Santos, Juliane F. S. Zibrandtsen, Disan Gunbilig, Mette Sørensen, Federico Cozzi, Berin A. Boughton, Allison Maree Heskes, Elizabeth Heather Jakobsen Neilson

**Affiliations:** ^1^Section for Plant Biochemistry, Department of Plant and Environmental Sciences, University of Copenhagen, Copenhagen, Denmark; ^2^VILLUM Center for Plant Plasticity, Department of Plant and Environmental Sciences, University of Copenhagen, Copenhagen, Denmark; ^3^Section for Molecular Plant Biology, Department of Plant and Environmental Sciences, University of Copenhagen, Copenhagen, Denmark; ^4^School of BioSciences, University of Melbourne, Parkville, VIC, Australia; ^5^Metabolomics Australia, School of BioSciences, University of Melbourne, Parkville, VIC, Australia; ^6^Center for Synthetic Biology ‘bioSYNergy’, Department of Plant and Environmental Sciences, University of Copenhagen, Copenhagen, Denmark

**Keywords:** *Corymbia*, *Eucalyptus*, formylated phloroglucinol compounds, macrocarpal, MALDI-mass spectrometry imaging, sideroxylonal, specialized metabolites

## Error in Figure/Table

In the original article, there was a mistake in **Figure 4** and **Supplementary Table S2** as published. There was an error during the FPCs quantification process, whereby the ratio of injection volume between sample and standard was accidentally inverted. This error has resulted in the overestimation of FPCs concentration reported, but does not alter the biological significance of the results. The corrected **Figure 4** appears below, and **Supplementary Table S2** has been replaced in the original article.

Furthermore, in the original article, there was an error in the results section where the number of total FPCs for different tissues of two species are cited.

A correction has been made to the *Results* section, sub-section *Detection and Quantification of FPCs*, paragraph four:

“From all species analyzed, *E. camphora* and *E. globulus* had the highest concentration of total FPCs in leaves, with *65* and *41*mg g−1 DW, respectively (**Figure 4**, **Supplementary Table S2**). *Eucalyptus camphora* also had high concentration of FPCs in flower buds and flowers, with *13* and *12*mg g−1 DW, respectively. Interestingly, three *Eucalyptus* species showed a tendency to accumulate more FPCs in flowers compared to the leaves. *Eucalyptus leucoxylon*, *E. sideroxylon*, and *E. viminalis* contained ~40, 5, and 3 times more total FPCs in the flowers compared to leaves, respectively **Figure 4**, **Supplementary Table S2**. *Eucalyptus yarraensis* presented very low amounts of FPCs in leaves and flower buds, and it is the only species that does not contain any sideroxylonals. *Eucalyptus cladocalyx* and *C. ficifolia* did not show any traces of this class of specialized metabolites in the tissues analyzed.”

**Figure 4:**
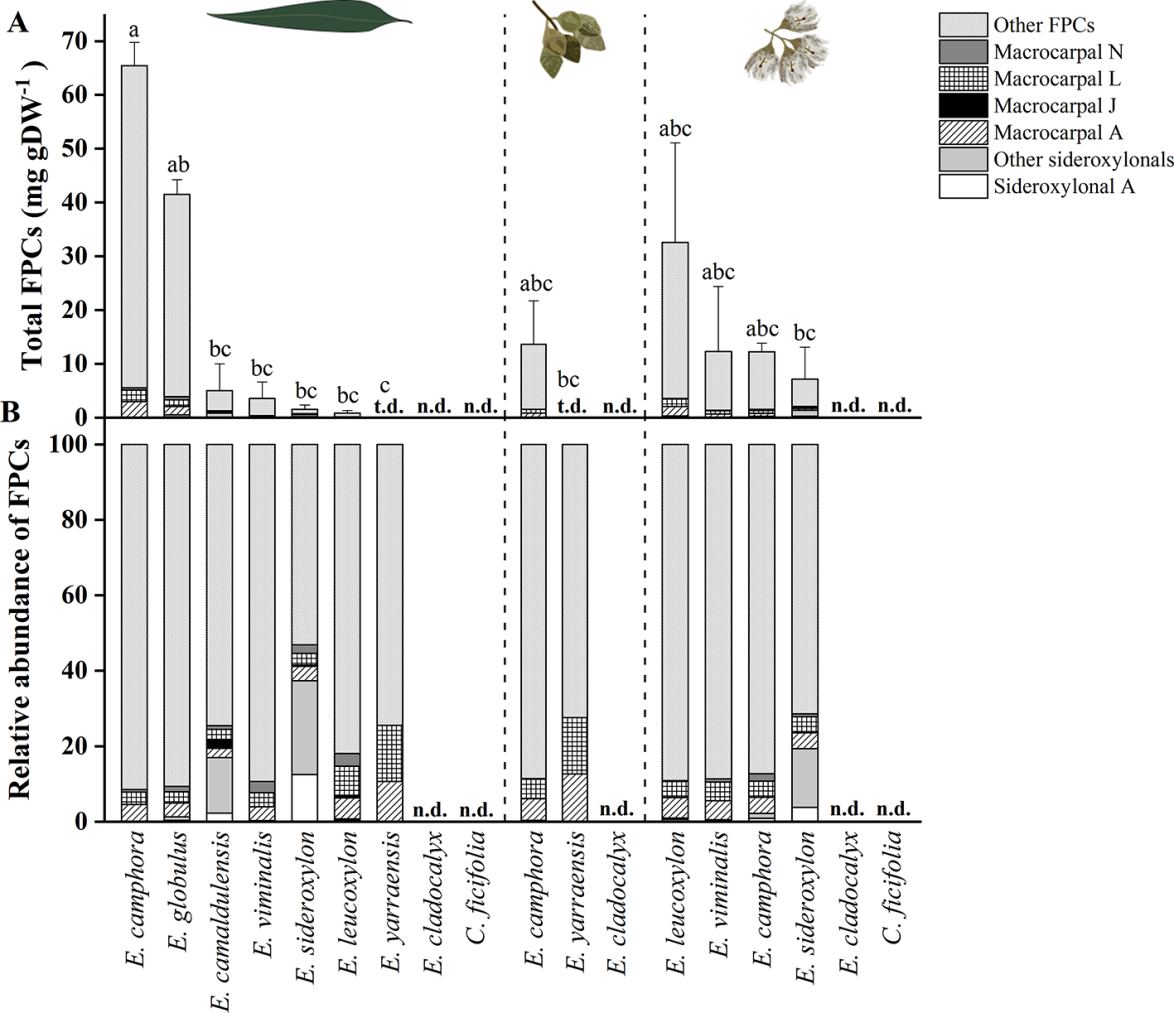
**(A)** Total FPCs concentration in leaves, flower buds and flowers of different eucalypt species. Bars represent mean ± standard error. Small letters represent statistical differences according to one-way ANOVA p < 0.050. **(B)** Relative FPCs concentration as percentage in respective tissues. t.d., traces detected; n.d., not detected.

In addition, there was an error in the discussion where the number of total FPCs concentration is cited again.

A correction has been made to the *Discussion* section, sub-section *Qualitative and Quantitative FPCs Variation in Eucalyptus*, paragraph three:

“*Eucalyptus camphora* and *E. globulus* presented high concentrations of total FPCs in expanded leaves, with *65* and *41* mg g−1 DW, respectively. *These concentrations are in a similar range to previous reports*. *For example*, the concentration of sideroxylonals have been reported to reach up to 52 mg g^−1^ DW in *E. melliodora* (Wallis et al., 2002) and up to 100 mg g^−1^ DW in *E. loxophleba* ssp. *lissophloia* (Wallis and Foley, 2005).”

The authors apologize for this error and state that this does not change the scientific conclusions of the article in any way. The original article has been updated.
